# Toward a More Nuanced View on Organizational Support Theory

**DOI:** 10.3389/fpsyg.2020.00476

**Published:** 2020-03-31

**Authors:** Gaëtane Caesens, Florence Stinglhamber

**Affiliations:** Psychological Sciences Research Institute, Université Catholique de Louvain, Louvain-la-Neuve, Belgium

**Keywords:** perceived organizational support, dark side, curvilinear relationships, moderators, organizational support theory

## Introduction

The perceived organizational support (POS) construct occupies a prominent place in organizational psychology and management literature, with more than 1,200 studies on this topic. Drawing on social exchange theory (Blau, 1964) and the norm of reciprocity (Gouldner, 1960), organizational support theory holds that by providing positive resources to employees, POS induces among employees a felt obligation to help the organization to reach its goals (Eisenberger et al., 1986; Rhoades and Eisenberger, 2002; Kurtessis et al., 2017). Additionally, organizational support theory assumes that POS fulfills important socioemotional needs in the workplace such as employee need for affiliation or approval, leading to self-enhancement processes (e.g., Eisenberger and Stinglhamber, 2011; Caesens et al., 2017). These two mechanisms (i.e., social exchange and self-enhancement) are the basis for explaining why a high POS leads to beneficial outcomes for both organizations and individuals (e.g., Eisenberger and Stinglhamber, 2011). Empirical evidence has indeed indicated that POS is related to a plethora of positive consequences, which have been classified into three main categories of outcomes, i.e., (1) employees' subjective well-being (e.g., increased job satisfaction, reduced burnout), (2) employees' positive orientation toward the organization and work (e.g., increased affective commitment, organizational identification, work engagement), and (3) employees' favorable behaviors (e.g., increased performance, and reduced absenteeism and turnover) (e.g., Rhoades and Eisenberger, 2002; Eisenberger and Stinglhamber, 2011; Kurtessis et al., 2017).

So far, the literature has thus mainly considered POS as a positive element in the workplace, having beneficial effects for both employees and organizations (Eisenberger and Stinglhamber, 2011). This theoretical opinion paper aims to shed new and more nuanced light on organizational support theory by considering the possibility that, in some cases, POS might cease to generate positive effects or even lead to aversive consequences. So far, the evidence of a potential dark side of POS is sparse, uncertain, and little discussed. Yet, there is some preliminary evidence that indicates that POS might have negative consequences for both organizations and employees under certain circumstances.

First, Armeli et al. (1998) unexpectedly discovered in their empirical study that, for workers having low socioemotional needs, the link between POS and performance was interestingly negative. The authors explained this finding by “the possibility that employees with low socioemotional needs may view high POS as a bank of good will that provides an opportunity to rest on one's laurels” (Armeli et al., 1998, p. 295). This unexpected result might also be understood in light of the threat-to-self-esteem model (Deelstra et al., 2003) which suggests that for employees having low socio-emotional needs, a high level of POS may be perceived negatively. In particular, a high POS could be perceived by employees as an indication of incompetence or a lack of confidence on the part of the organization toward them, leading to a reduction of employee performance.

Second, based on the “too-much of a good thing effect” (TMGT effect) proposed in the management literature (Pierce and Aguinis, 2013; Burnett et al., 2015) demonstrated that the relationship between POS and employees' taking charge is characterized by a curvilinear inverted U-shaped curve. They suggested that when good things are present in excess and reach a threshold (e.g., supportiveness) it can backfire. More precisely, Burnett et al. (2015) integrated the threat-to-self-esteem model (Nadler and Fisher, 1986) and the social exchange theory (Gouldner, 1960) to explain this curvilinear effect. They proposed that, when POS is present in excess, it can be perceived as self-threatening (employees may perceive POS as an indication of being incompetent or overhelped) and thus employees become unable to reciprocate for a high level of POS, resulting in subsequent negative reactions. Interestingly, this curvilinear relationship was also found to be stronger for employees having high concerns or perceiving high anticipated costs related to a potential non-receptivity to their proactive behavior.

Third, again based on the TMGT effect (Pierce and Aguinis, 2013; Harris and Kacmar, 2018) recently proposed that employees perceiving high levels of support might at some point consider that they have done enough efforts to reciprocate for the positive treatments received from the organization, leading to plateauing effect in the POS-outcomes relationships that can either lead to asymptotic (nor more beneficial effect) or negative effects. In line with this, they found that the relationships between POS and in-role performance, extra-role performance, affective commitment and deviance (all rated by supervisors) were non-linear (characterized by an inverted *U*-shaped curve for in—role, extra-role performance, and affective commitment, and a *U*-shaped curve for deviance). Unfortunately, these scholars did not explicitly test whether the nonlinear relationships found lead to a ceiling effect or to actual negative effects after the inflection point. They concluded that the overall shape of the relationships between POS, in-role, extra role performance, and affective commitment were characterized by “upwardsloping lines until a certain point (the inflection point) where the line no longer slopes upward and even goes down at the end” (p. 194).

## Future Lines OF Research

Based on this prior first evidence, it seems relevant to explore in the future years the conditions under which POS might lead to negative consequences, or at least to cease to have beneficial effects in order to offer a better comprehension of the POS construct. We propose here several directions for future research that can be explored.

First, research on the curvilinear effects of POS should be continued. To date, this research has focused on consequences in terms of positive orientation (e.g., affective commitment; Harris and Kacmar, 2018) or favorable work behaviors (e.g., proactivity, performance; Burnett et al., 2015; Harris and Kacmar, 2018). These curvilinear effects were mainly explained through the equity theory (Adams, 1965) which is at the core of social exchange and suggests that the benefits received by one party should be proportional to investments made by the other party. An unbalance in this reciprocity (to receive more than what we give) might lead to iniquity feelings, generating negative emotions among employees (Scheer et al., 2003). While cultural differences may mitigate these effects (e.g., Scheer et al., 2003), an excessive level of POS might lead to an asymmetric relationship of inequity where an employee does not feel able or does not want to reciprocate, which would reduce his/her feeling of obligation toward the organization and his/her subsequent positive attitudes or behaviors toward the organization. Even less is known regarding the consequences of an excessive level of POS on employees' subjective well-being. Yet, as described earlier, some theories related to the threat-to-self-esteem model (Deelstra et al., 2003) suggest that an excessive level of POS could induce among employees a feeling of incompetence and limitation of freedom and autonomy, which could influence their well-being. In line with this, we suggest that at some extreme level, too much POS could lead to detrimental consequences for employee well-being such as increased stress levels or reduced job satisfaction. Future research should examine these potential nonlinear associations between POS and various outcomes, particularly employees' well-being, while providing a sound theoretical framework and explanations for such effects. In this perspective, we encourage researchers to further examine whether they found a ceiling effect of POS or actual adverse effects after the inflection point and to investigate the reasons for such effects.

Second, it might be that situational individual factors reverse the relationship between POS and beneficial consequences (or exacerbate the nonlinear effects, see prior point). These potential moderators of POS certainly need further investigation. For instance, for employees who are highly perfectionist, a high level of POS might lead to higher levels of stress as they feel that they have to reciprocate for the high level of support received. Similarly, for employees having high equilibrium in exchange (i.e., high in equity sensitivity), a high level of POS might lead to perceive a particular imbalance in the reciprocity, leading to more negative subsequent consequences. In another vein, negative social comparisons occurring between employees regarding their assessment of POS (i.e., perceiving to be less supported by the organization as compared to colleagues) might also lead to less beneficial outcomes. Finally, low perceived organizational competence (Kim et al., 2016) or low organizational prestige (Marique et al., 2013) might also exacerbate the potential nonlinear effects of POS on outcomes.

Third, future research should examine the dynamic of POS over time using longitudinal designs and multiple measurement time. Indeed, most prior research considered POS as a stable variable. Yet, it may be relevant to examine this construct dynamically and to analyze whether employees who generally perceive high levels POS may react more negatively to a slight decrease in their POS over time, as compared to employees who are characterized by stable perceptions of moderate support from their organization over time. This suggests that providing a high POS could generate negative consequences if it is not fully maintained, and thus provide interesting insight regarding its effects.

## Conclusion

Overall, this future line of research will certainly contribute to a more nuanced perspective on organizational support theory (e.g., Eisenberger and Stinglhamber, 2011; Kurtessis et al., 2017), by providing a better comprehension of the effects of POS and will also help managers to optimize their allocation of supportive resources.

## Author Contributions

GC and FS wrote the paper. All authors contributed to manuscript, read, and approved the submitted version.

### Conflict of Interest

The authors declare that the research was conducted in the absence of any commercial or financial relationships that could be construed as a potential conflict of interest.

## References

[B1] Adams J. S. (1965). “Inequity in social exchange,” in Advances in Experimental Social Psychology, ed L. Berkowitz (New York, NY: Academic Press) 267–299. 10.1016/S0065-2601(08)60108-2

[B2] Armeli S. Eisenberger R. Fasolo P. Lynch P. (1998). Perceived organizational support and police performance: the moderating influence of socioemotional needs. J. Appl. Psychol. 83, 288–297. 10.1037/0021-9010.83.2.288 9577236

[B3] Blau P. M. (1964). Exchange and Power in Social Life. New York, NY: Wiley.

[B4] Burnett F. B. Chiaburu D. S. Shapiro D. L. Li N. (2015). Revisiting how and when perceived organizational support enhances taking charge: an inverted U-shaped perspective. J. Manage. 41, 1805–1826. 10.1177/0149206313493324

[B5] Caesens G. Stinglhamber F. Demoulin S. De Wilde M. (2017). Perceived organizational support and employees' well-being: the mediating role of organizational dehumanization. Eur. J. Work Organiz. Psychol. 26, 527–540. 10.1080/1359432X.2017.1319817

[B6] Deelstra J. T. Peeters M. C. W. Zijlstra F. R. H. Schaufeli W. B. Stroebe W. van Doornen L. P. (2003). Receiving instrumental support at work: when help is not welcome. J. Appl. Psychol. 88, 324–331. 10.1037/0021-9010.88.2.324 12731716

[B7] Eisenberger R. Huntington R. Hutchison S. Sowa D. (1986). Perceived organizational support. J. Appl. Psychol. 71, 500–507. 10.1037/0021-9010.71.3.500

[B8] Eisenberger R. Stinglhamber F. (2011). Perceived Organizational Support: Fostering Enthusiastic and Productive Employees. Washington, DC: APA Books 10.1037/12318-000

[B9] Gouldner A. W. (1960). The norm of reciprocity: a preliminary statement. Am. Soc. Rev. 25, 161–178. 10.2307/2092623

[B10] Harris K. J. Kacmar K. M. (2018). Is more always better? An examination of the nonlinear effects of perceived organizational support on individual outcomes. J. Soc. Psychol. 158, 187–200. 10.1080/00224545.2017.1324394 28481736

[B11] Kim K. Y. Eisenberger R. Baik K. (2016). Perceived organizational support and affective organizational commitment: moderating influence of perceived organizational competence. J. Organ. Behav. 37, 558–583. 10.1002/job.2081

[B12] Kurtessis J. N. Eisenberger R. Ford M. T. Buffardi L. C. Stewart K. A. Adis C. S. (2017). Perceived organizational support: a meta-analytic evaluation of organizational support theory. J. Manage. 43, 1854–1884. 10.1177/0149206315575554

[B13] Marique G. Stinglhamber F. Desmette D. Caesens G. De Zanet F. (2013). The relationship between perceived organizational support and affective commitment: a social identity perspective. Group Organiz. Manage. 38, 68–100. 10.1177/1059601112457200

[B14] Nadler A. Fisher J. D. (1986). The role of threat to self-esteem and perceived control in recipient reaction to help: theory development and empirical validation. Adv. Exp. Soc. Psychol. 19, 81–122. 10.1016/S0065-2601(08)60213-0

[B15] Pierce J. R. Aguinis H. (2013). The too-much-of-a-good-thing effect in management. J. Manage. 39, 313–338. 10.1177/0149206311410060

[B16] Rhoades L. Eisenberger R. (2002). Perceived organizational support: a review of the literature. J. Appl. Psychol. 87, 698–714. 10.1037/0021-9010.87.4.698 12184574

[B17] Scheer L. K. Kumar N. Steenkamp J. E. (2003). Reactions to perceived inequity in US and Dutch interorganizational relationships. Acad. Manage. J. 46, 303–316. 10.2307/30040624

